# P-1339. Navigating Antibiotic Susceptibility Testing: The Antibiotic Breakpoint Explorer (ABE 1.0)

**DOI:** 10.1093/ofid/ofae631.1516

**Published:** 2025-01-29

**Authors:** Arryn Craney, Suetping Lau, Addae Jones, Sanjay Mandal

**Affiliations:** MiraVista Diagnostics, Orlando, Florida; Petrified Bugs / Orlando Health, Orlando, Florida; Petrified Bugs, Indianapolis, Indiana; Petrified Bugs, Indianapolis, Indiana

## Abstract

**Background:**

The landscape of antibiotic susceptibility testing has evolved significantly, with constant revisions to antibiotic breakpoint definitions by key standard-setting bodies: the Clinical and Laboratory Standards Institute (CLSI), the Food and Drug Administration (FDA), and the European Committee on Antimicrobial Susceptibility Testing (EUCAST). These changes reflect advances in scientific understanding, emerging resistance patterns, and the need for harmonization across international guidelines. However, staying abreast of these evolving breakpoints can be challenging for healthcare professionals. The Antibiotic Breakpoint Explorer 1.0 (ABE 1.0) is an innovative tool that simplifies antibiotic susceptibility testing interpretation with current 2024 breakpoint interpretive criteria.

**Figure:**
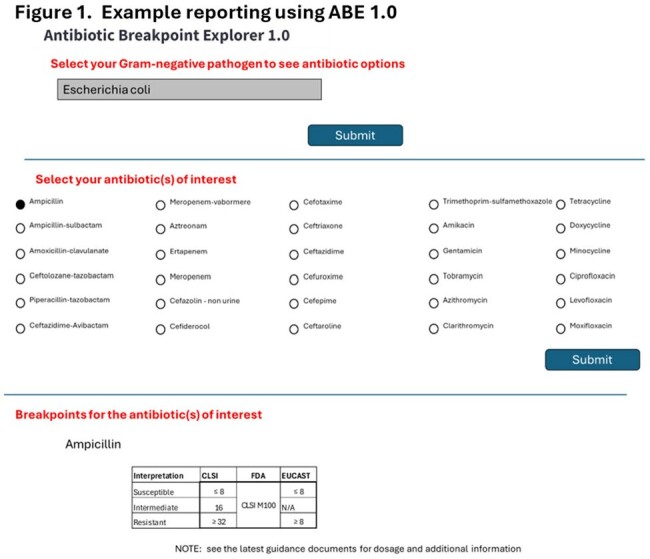

**Methods:**

Antibiotic breakpoints for minimum inhibitor concentration (MIC) were consolidated from the CLSI, FDA and EUCAST websites for current 2024 breakpoint interpretative criteria for 15 Gram-negative pathogens and 30 antibiotics from 10 antibiotic classes (Table 1). ABE 1.0 was programmed using Python 3.12.2 and the Streamlit package. ABE 1.0 is hosted as a web-based tool hosted at www.petrifiedbugs.com. The initial beta version of the program is accessible at https://newbreak-hxdfezfsrkpuusfjpfkrby.streamlit.app/

**Figure:**
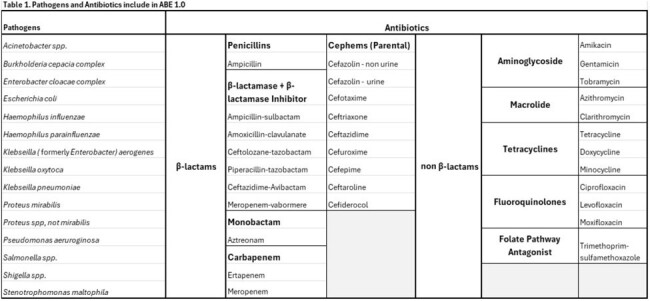

**Results:**

By consolidating breakpoint information from CLSI, FDA, and EUCAST guidelines for the top 15 Gram-negative pathogens, ABE 1.0 provides a simple user-friendly web-based tool to help users make informed decisions regarding antibiotic therapy and resistance management for 30 key clinically used antibiotics (Figure 1).

**Conclusion:**

ABE 1.0 addresses the challenge of navigating antibiotic breakpoints by offering a user-friendly platform that consolidates antibiotic breakpoint information from CLSI, FDA, and EUCAST guidelines into a single, easily accessible resource.

**Disclosures:**

**Arryn Craney, PhD D(ABMM)**, MiraVista Diagnostics: Employee; Salary

